# The impact of challenge and hindrance stressors on newcomers’ organizational socialization: A moderated-mediation model

**DOI:** 10.3389/fpsyg.2022.968852

**Published:** 2022-09-28

**Authors:** Yi Tang, Zhijun Zhang, Shengnan Wu, Ju Zhou

**Affiliations:** ^1^Chongqing Academy of Governance, Chongqing, China; ^2^Department of Psychology and Behavioral Sciences, Zhejiang University, Hangzhou, China

**Keywords:** challenge stressors, hindrance stressors, job crafting, leader-member exchange, organizational socialization

## Abstract

The importance of work stress on newcomers’ organizational socialization has been a topic of substantial interest. However, the impact mechanism of different stressors on newcomers’ organizational socialization is still in the early stages of theory development. This study, based on the theory of the dual work stress model, explored how to challenge stressors and hindrance stressors impact newcomers’ organizational socialization *via* the mediation of job crafting. Based on the empirical data from 247 newcomers, we found that challenge stressors positively affected newcomers’ organizational socialization; on the contrary, hindrance stressors would result in negative influences. In addition, leader-member exchange enhanced the positive effect of challenge stressors on newcomers’ job crafting and further moderated the indirect influence of challenge stressors on newcomers’ organizational socialization *via* job crafting. These findings provide a practical guide for organizations to apply stress management and promote newcomers’ socialization.

## Introduction

After a newcomer enters an organization, it takes a period to adapt to the new environment and become familiar with the new job, changing from an outsider to an insider. This process is called the process of organizational socialization. Researchers believe that ineffective organizational socialization is one of the main factors that cause newcomers to quit or be fired (Fisher, 1986), which would increase the cost of the organization in terms of talent acquisition, training, and mobility (Kammeyer-Mueller and Wanberg, 2003; Bodoh, 2012). Many present studies from the labor market show that employees are more likely to leave (e.g., Farber, 1994), especially young employees, whose tenure in their first job is shortening year by year. Some researchers also have proposed that in the context of increasing newcomer mobility, efficient organizational socialization would play a greater role (Chen and Klimoski, 2003). In recent years, researchers have attempted to explain organizational socialization processes from different theoretical perspectives (Feldman, 1981; Saks and Ashforth, 1997; Kammeyer-Mueller and Wanberg, 2003): from the impact of newcomer characteristics on organizational socialization (Jones, 1983; Nicholson, 1984; Miller and Jablin, 1991; Alessandri et al., 2018), the organizational socialization strategy adopted by the organization (Van Maanen and Schein, 1979; Benson and Eys, 2017), the impact of the interaction between new and old employees on organizational socialization (Reichers, 1987; Moreland and Levine, 2001; Ashforth et al., 2016; Ellis et al., 2017), and the relationship between stress and organizational socialization (Berger and Calabrese, 1975).

The new work environment is full of stress for newcomers. Organizational socialization is considered a process for newcomers to reduce the stress caused by uncertainty (Berger and Calabrese, 1975), which indicates that work stress plays an important role in the newcomers’ socialization in organizations. Saks and Gruman (2012) believed that it is very important to understand newcomers’ organizational socialization from the perspective of stress, which can help newcomers reduce possible losses in adapting to the new environment. Many researchers have also tried to examine the relationship between work stress and organizational socialization (Nelson and Sutton, 1990; Bravo et al., 2003; Liang and Hsieh, 2008), but few empirical research results have been obtained. Some researchers believe that although researchers have realized that it is necessary to explore the role of stress in the process of organizational socialization many years ago, the progress of Research in recent years is still slow (Ellis et al., 2015). The development of stress theory in recent years provides a more in-depth theoretical perspective for studying the relationship between stress and organizational socialization. Some early views on work stress generally believed that work stress would only have a negative impact on employees’ work behaviors, such as reducing employees’ enthusiasm for work and causing counterproductive behaviors. However, the Research of Hartline and Ferrell (1996) shows that work stress can improve employees’ positive work behaviors, such as job satisfaction and work engagement.

Therefore, researchers have proposed a two-dimensional structure theory of stress according to employees’ different experiences of stress (Cavanaugh et al., 2000; Lu et al., 2010). They classified stressors as challenge stressors and hindrance stressors. Challenge stressors refer to job demands beneficial to an employee’s work level and career development and are generally considered “good” stress. These job requirements include a high workload, time pressure, and high levels of job responsibility (Cavanaugh et al., 2000; Crawford et al., 2010), which can generate future employee benefits (Rodell and Judge, 2009). Once newcomers overcome these pressures, they will have a higher sense of accomplishment, activate a stronger willingness to integrate and learn and induce positive job performance, such as increased job performance, job satisfaction, and organizational loyalty. In contrast, hindrance stressors are “bad” stress and difficult to overcome. They will inhibit employee work behavior. Examples of hindrances include demands such as role ambiguity, organizational policies, red tape, and hassles. They can reduce employee motivation, leading to negative job performance such as turnover intentions, anti-productive and withdrawn behaviors, etc. (Boswell et al., 2004; Podsakoff et al., 2007). Research shows that challenge stressors stimulate positive work behaviors and emotions, while hindrance stressors encourage negative work behaviors and emotions (Lepine et al., 2005). This dichotomy classification of stressors has proven to be a promising classification method by other scholars (O'Brien and Beehr, 2019). There may be differences in the impact mechanism of different types of stressors on outcome variables (Podsakoff et al., 2007). However, whether and how the challenge and hindrance stressors will influence newcomers’ organizational socialization is still a “black box” so far. Thus, the current study aims to fill in these gaps and examine how two types of stressors shape newcomers’ organizational socialization based on the challenge-hindrance stressor model.

Furthermore, the study hypothesized that the process and potential explanation of the impact of different challenges versus hindrances on organizational socialization would also depend on other workplace resources. Specifically, this study considered LMX quality as a potential moderating variable. It is often seen as a key prerequisite for employees to have or not to have diverse and influential work resources (Graen and Uhl-Bien, 1995; Dulebohn et al., 2012) and may also affect challenge versus hindrance stressor-related processes (Montani et al., 2017; Chen and Fang, 2019). Researchers have argued that LMX can be considered a workplace resource that occupies a primary position in the JD-R model (Loi et al., 2011). For example, it has been shown that employees with good LMX relationships experience lower levels of role ambiguity and conflict (Dulebohn et al., 2012), while newcomers’ role perceptions are closely related to organizational socialization. Furthermore, LMX is a highly malleable environmental factor (van den Heuvel et al., 2015), and the selection of LMX as a potential moderator is theoretically relevant and practically useful.

This study expands the extant literature and aims to make at least three contributions. First, we illustrate the different impacts of challenge and hindrance stressors on newcomers to the extent of the research on socialization. Organizational entry is high-pressure for newcomers, but such experiences do not always prohibit newcomers from adjusting successfully. As challenge and hindrance stressors affect individuals’ work behaviors, comprehending different stressors in the newcomer context is significant. Second, this study advances the work stress literature by exploring whether and how stressors impact newcomers’ socialization based on the challenge-hindrance stressor model. Third, this study extends the job demands-resources theory by revealing the mediating role of job crafting and moderating role of leader-member exchange. From the perspective of job resources, this study provides a new theoretical perspective and explains how stressors impact newcomers’ socialization *via* individual strengths and environmental resources.

## Theory and hypothesis

### The influence mechanism of challenge stressors and hindrance stressors on newcomer’s organizational socialization

When newcomers enter the organization in the early stage, they are often accompanied by various high-pressure states such as uncertainty of professional competence, insufficient information, and new interpersonal interactions (Firth et al., 2014; Ellis et al., 2015), which may easily produce many negative emotional experiences such as anxiety (Kammeyer-Mueller et al., 2012). In the past 30 years, many researchers have believed that it is necessary to understand and explain the organizational socialization of newcomers from the perspective of stress (Nelson, 1987; Saks and Gruman, 2012). Berger and Calabrese (1975) proposed the uncertainty reduction theory and argue that uncertainty in job tasks, roles, and social relationships in a new work environment can create stress for newcomers (Jackson et al., 1987). Providing newcomers with information to help them enhance learning and effectively reduce uncertainty (Klein and Heuser, 2008) or stress (Wang et al., 2015) can help their organizational socialization. Empirical studies have also found that certain pressures (such as role pressure, including role conflict or role ambiguity, etc.) are important indicators for examining the socialization effect of newcomer organizations (Bauer et al., 1998; Saks et al., 2007). Therefore, further empirical investigation of what role job stress plays in the organizational socialization of newcomers would be of great significance.

Organizational socialization refers to the transformation of newcomers from “outsiders” to “insiders” after they enter the organization (Bauer et al., 2007). According to the multilevel process model proposed by Saks and Ashforth (1997), the outcomes of organizational socialization can be divided into proximal and distal outcomes, including organizational commitment, organizational citizenship behavior, performance, role innovation, etc., and lower absenteeism and turnover rates (Yao and Yue, 2008). Currently, researchers have conducted fewer direct studies on the perceived stress of newcomers and their organizational socialization outcomes, and have focused more on examining the effects of various stresses on a particular organizational behavior that is closely related to organizational socialization outcomes. For example, researchers have examined the effects of various stressors on employees’ proactive career management (Liu and Li, 2018), worker-organizational identity (Liu J. et al., 2019; Zhou and Ning, 2020), organizational commitment (Siu, 2003; Li et al., 2021), organizational citizenship behavior (Pooja et al., 2016), and turnover intentions (Han et al., 2015), and other influential roles of organizational behaviors related to organizational socialization outcomes. Studies using the challenge-hindrance stressor structure show that challenge stressors significantly and positively influence certain organizational behaviors related to organizational socialization outcomes (e.g., role identification, Li et al., 2018; Work Well-being, Liu Y. et al., 2019). However, hindrance stressors are the opposite. Hindrance stressors (role conflict and role ambiguity) can significantly negatively affect organizational behaviors related to organizational socialization outcomes such as job satisfaction and organizational commitment, and significantly positively affect the propensity to leave (Antón, 2009). However, it has also been shown that both challenge stressors and hindrance stressors significantly and positively predict job burnout (Wu et al., 2017). Work burnout is closely related to ineffective organizational socialization. To sum up, this study argues that there are differences in the impact of challenge stressors and hindrance stressors on newcomer’s organizational socialization after entry.

Therefore, the following hypotheses were proposed:

*H1a*: Challenge stressors positively affect the organizational socialization of new newcomers.

*H1b*: Hindrance stressors negatively affect the organizational socialization of new newcomers.

### The mediating role of job crafting

Wrzesniewski and Dutton (2001) formally proposed the concept of job crafting. Job crafting means that employees will autonomously change and shape work tasks, relationships, and cognition from the bottom up according to their own needs to obtain a higher sense of work meaning and identity. Tims and Bakker (2010), in conjunction with the job demands-resources theory (JD-R; Demerouti et al., 2001), define job crafting as a series of changes that employees make to match better their abilities, needs, and performance with their jobs based on their job demands and resources. According to the JD-R model, job demands refer to the physical and psychological demands of the job, such as high work load, time pressure, and interpersonal interactions. On the other hand, job resources are those elements of the job that help employees achieve their job goals, reduce job demands, and promote personal growth, such as job skills, leadership support, and learning opportunities (Bakker and Demerouti, 2007). The JD-R model suggests that the interaction between specific job demands and specific job resources affects employees’ job wellbeing. High job demands and low job resources can trigger burnout or turnover, but increasing challenge job demands and resources lead to better job outcomes (Bakker and Demerouti, 2007). Thus, newcomers encounter job requirements in the JD-R framework by job crafting to increase their job adaptability, for example, increasing structural job resources and social work resources (e.g., learning new knowledge, seeking feedback from leaders, etc.), seeking more challenging demands (e.g., joining in new projects), or taking the initiative to reduce certain work demands, such as reducing contact with more bureaucratic colleagues. Research has shown that the complexity and challenge of tasks (Berg et al., 2010) and challenge stressors (Harju et al., 2016) would increase job-crafting behaviors. The career dynamics model (proposed by Fried et al.) suggests that employees are more likely to engage in job crafting in the early stages of their careers (Fried et al., 2007), and job crafting has a positive effect on employees’ ability to work sustainably (Kira et al., 2010). The job-crafting framework developed by Tims et al. (2012) includes increasing structural job resources, decreasing hindering job demands, and increasing social jobs. This means that when newcomers are faced with challenge stressors, they may be automatically motivated to reinvent their jobs and even develop more challenge demands based on existing challenge stressors. In contrast, when newcomers are exposed to hindrance stressors, they are required to seek more job resources to reduce hindrance job demands, resulting in lower willingness to reinvent their jobs. Previous studies have shown that there was a significantly positive relationship between challenge stressors and job crafting, while hindrance stressors were negatively related to job crafting (Liu Y. et al., 2019; Chen et al., 2021). In addition, studies also show that challenge stressors have a significant negative impact on job crafting (Liu and Zhao, 2019).

Therefore, the following hypotheses were proposed:

*H2a*: Challenge stressors have a significant positive effect on job crafting.

*H2b*: Hindrance stressors have a significant negative effect on job crafting.

Newcomers need to take proactive behaviors in the early stages of entering the organization to reduce anxiety, improve their wellbeing (Cooper-Thomas and Wilson, 2011), and speed up the process of organizational socialization to increase their work experience (Ashford and Black, 1996). Cooper-Thomas et al. (2012) have shown that newcomers promote their organizational socialization process by changing roles or circumstances (such as changing work procedures; Cooper-Thomas et al., 2012). At the same time, job crafting can significantly improve the internal perception of newcomers (Cheng et al., 2019). Based on this, combined with Tims and Bakker's (2010) job-crafting framework based on the JD-R model, challenge stressors are job requirement for newcomers. However, job crafting can transform challenge job demands into important job resources. We can get that when newcomers are faced with challenge stressors, they can completely improve their job adaptability skills through job crafting and transform themselves into organizational “insiders” as soon as possible. This means that their organizational socialization is enhanced. At the same time, hindrance stressors are also job demands. Newcomers can reduce these demands through job crafting to help them adapt better to the work environment. However, this process requires additional work resources. Therefore, when newcomers are faced with hindrance stressors, they cannot promote their organizational socialization exclusively through job crafting. Therefore, the following hypotheses were proposed:

*H3a*: Job crafting will completely mediate the relationship between challenge stressors and organizational socialization.

*H3b*: Job crafting will partially mediate the relationship between hindrance stressors and organizational socialization.

### The moderating role of leader-member exchange

Leader-member exchange (LMX) was first proposed by Graen et al., 1972, who believed that leaders would develop different relationships with different members due to their limited time, resources, and energy. The members with high-quality LMX exchange gradually evolved into “in-group members, “and those with low-quality exchange developed into “out-group members.” Leaders establish a social exchange with “in-group members” based on mutual reciprocity, trust, and respect and maintain an economic exchange with “out-group members” within the scope of work and contractual requirements (Graen and Uhl-Bien, 1995; Sparrowe and Liden, 1997). LMX is considered the most important and formal interpersonal relationship among employees’ many social network relationships (Chong et al., 2015) and is closely related to the organizational socialization process of newcomers (Graen and Cashman, 1975; Graen and Uhl-Bien, 1995). Research has shown that the higher quality of LMX, the more resources, information, empowerment, etc., members receive (Dienesch and Liden, 1986). Employees who perceive low LMX might receive little support or resources from their leaders (Graen and Uhl-Bien, 1995). They rely primarily on personal resources to cope with the stressors they face. Based on the conservation of resources theory, an individual’s personal resources are limited. The ability to cope with challenge stressors is also limited (Hobfoll, 1989), and having more leadership support means that newcomers have access to more external resources, and these resources from leaders are an important way for them to job crafting (e.g., increasing social job resources, Tims et al., 2012). Therefore, when employees face lower challenge stressors, they have enough personal resources to cope with these stressors. As a result, the high or low LMX does not affect their job crafting, but when the challenge stressors they face are large enough, newcomers who perceive low LMX may feel a lack of additional resources for job crafting. In contrast, newcomers who perceive high LMX are then able to undertake more job crafting. Researchers believe that resources from leaders are an important contingency factor that affects employees’ response to challenge stressors (Lian et al., 2012). It has been demonstrated that employees with higher LMX are more willing to devote ample resources and show more scope for self-expression when faced with challenge stressors (Scott and Bruce, 1994). The study by Spurk et al. (2021) showed that LMX could moderate the relationships between competitive psychological climate (CPC) and work engagement (CPC appraised as challenge) and burnout (CPC appraised as hindrance). Therefore, the following hypotheses were proposed:

*H4a*: LMX moderates the relationship between challenge stressors and job crafting, and the moderating effect further predicts organizational socialization of newcomers through job crafting.

*H4b*: LMX moderates the relationship between hindrance stressors and job crafting, and the moderating effect further predicts organizational socialization of newcomers through job crafting.

**Figure 1:**
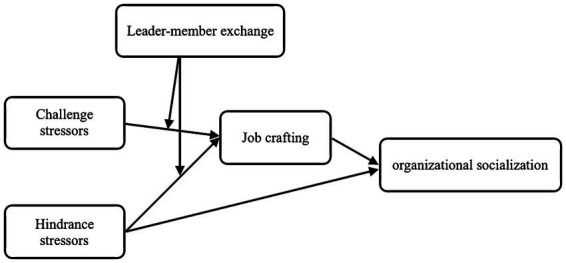
Conceptual model.

In conclusion, this study investigated the mechanism of challenge stressors-hindrance stressors on organizational socialization of newcomers by constructing a moderated mediation model. The theoretical model is shown in Figure 1.

## Materials and methods

### Data collection and sample selection

The participants in this study were newly recruited civil servants working in local government from one Chinese province. They came from 40 districts and counties, and each participant came from a different work unit. They received our on-site paper-based questionnaire while attending uniform vocational training at their school. We defined newcomers as those who have worked in their current position for about 1 year. The questionnaire design set the job duration option at 9 to 15 months. After obtaining permission from managers, we described the specifics of the survey to respondents and assured all participants that the study was voluntary, confidential, anonymous, and not related to their work or academic performance assessment. In addition, they were reminded of the importance of honest responses to academic research. The effect of homologation bias was reduced by adopting a balanced order of items and reducing participants’ guesswork about the purpose of the test. After participants completed the questionnaire, they submitted it directly to the research team.

A total of 270 questionnaires were distributed, and 247 questionnaires were finally valid, with an effective recovery rate of 91.48%. The distribution of valid samples is as follows: in terms of gender, men account for 50.60%, and women account for 49.40%; in terms of age, born between 1990 and 1994 account for 38.50%, born in 1995 or later account for 61.50%; and in terms of education, Master’s degree and above accounted for 51.80%.

### Variable measurement

The scale of challenge stressors-hindrance stressors. This study used the scale designed by Cavanaugh et al., 2000, with 11 items. The scale of challenge stress consisted of 6 items, and that of hindrance stressors consisted of 5 items. For example, “The number of projects and/or assignments I have” and “The amount of red tape I need to go through to get my job done.” Participants were asked to indicate their stress level during the job (e.g., 1 = no stress, 5 = great deal of stress). The scale has good measurement indicators and has been widely used by many researchers (Liu et al., 2011; Li and Li, 2013). Cronbach’s alpha coefficients for challenge stressors and hindrance stressors were 0.86 and 0.76, respectively.

The scale of Job crafting. This study used the Job-crafting Scale developed by Tims et al. (2012), with a total of 21 items. It included four dimensions: increasing structural job resources, decreasing hindering job demands, increasing social job resources, and increasing challenging job demands. An example item, “When an interesting project comes along, I offer myself proactively as a project co-worker.” Responses were given on a 5-point scale with 1 (totally disagree) to 5 (totally agree). Domestic researchers have confirmed that the scale was suitable for my country’s cultural context (Yin et al., 2016), with good reliability and validity. The Cronbach’s alpha coefficient for this scale was 0.91.

The scale of Leader-member exchange. This study adopted the leader-member exchange scale compiled by Wang et al. (2004), with a total of 16 items. It includes four dimensions emotion, loyalty, contribution, and professional respect. An example item, “I would be a pleasure to communicate with my supervisor.” Responses were given on a 5-point scale with 1 (totally disagree) to 5 (totally agree). The Cronbach’s alpha coefficient for this scale was 0.83.

The scale of Organizational socialization. This study used the Organizational Socialization Scale developed by Wang (2012). There are 20 items in total, including three dimensions: organizational culture socialization, interpersonal relationship socialization, and job competency socialization. An example item, “I know all the procedures necessary to get the job done successfully.” Responses were given on a 5-point scale with 1 (totally disagree) to 5 (totally agree). The Cronbach’s alpha coefficient for this scale was 0.90.

Control variables. Combined with the existing research results, this study included gender, age, and education as control variables in the model to better reflect the relationship between variables. This study used statistical software such as SPSS 25.0, PROCESS macro program, and AMOS 24.0 to process the data. Data analysis adopted various statistical methods such as reliability and validity analysis, descriptive statistics, correlation analysis, multiple regression analysis, and the bootstrap method.

## Results

### Validity test

In this study, AMOS 24.0 was used to test the overall fitness of the model. Firstly, the balance method in the packaging strategy (Wu and Wen, 2011) is used for packaging the constructs according to the factor load of the items. Each packaged construct contains four to six items. Second, five-factor, four-factor, three-factor, two-factor, and single-factor models were compared, respectively (Table 1). Table 1 shows that the five-factor model had a good fit (*χ2/df =* 2.17, *CFI =* 0.93, *TLI* = 0.92, *RMSEA* = 0.06), and was significantly better than other competing models. This result indicated that each variable in the model has good discriminant validity. The composite reliability values of all five dimensions, including challenge stressors, hindrance stressors, job crafting, leader-member exchange, and organizational socialization, are 0.89, 0.82, 0.90, 0.92, and 0.86, which are all greater than 0.6. The AVE values of the five constructs were all higher than 0.5, indicating that the scale had good convergence validity.

**Table 1:** Results of confirmatory factor analysis of measurement model.

Measurement model	*χ^2^*	*df*	*Δχ^2^*	*RMSEA*	*CFI*	*TLI*
Five-factor model (*X1, X2, M, W, Y*)	430.94	199		0.06	0.93	0.92
Four-factor model (*X1, X2, M* + *W, Y*)	943.88	203	512.95	0.12	0.78	0.75
Three-factor model (*X1* + *X2, M* + *W, Y*)	1162.51	206	218.63	0.14	0.71	0.68
Two-factor model (*X1* + *X2, M* + *W* + *Y*)	1487.13	208	324.62	0.16	0.62	0.57
One-factor model (*X1* + *X2 + M* + *W + Y*)	2133.94	209	655.80	0.19	0.42	0.36

*X1*, Challenge stressors; *X2*, Hindrance stressors; *M*, Job crafting; *W*, Leader-member exchange; *Y*, Organizational socialization.

### Common method variance

This study adopted the Harman single-factor test method after data collection (Zhou and Long, 2004) to avoid the common error variance by collecting multiple variable data in the self-assessment method. Without rotating the factors, the variance of the first factor is 23.38%, which does not exceed the 40% criterion. The results indicated that the common error variance of the questionnaire is acceptable. In addition, the fitting indices of the single-factor model were not qualified (*χ^2^/df* = 10.21, *CFI* = 0.42, *TLI* = 0.36, *RMSEA =* 0.19), indicating that the common error variance of the data is not large.

### Descriptive statistical analysis

The mean, standard deviation, and correlation coefficient matrix of each variable are shown in Table 2. Challenge stressors were significantly positively correlated with job crafting (*r* = 0.25, *p* < 0.01). Challenge stressors were significantly positively correlated with organizational socialization (*r* = 0.19, *p* < 0.01). Hindrance stressors were significantly negatively correlated with job crafting (*r* = −0.14, *p* < 0.05). Hindrance stressors were significantly negatively correlated with organizational socialization (*r* = −0.22, *p* < 0.01). Job crafting was positively correlated with organizational socialization (*r* = 0.54, *p* < 0.01). The above results have provided preliminary support for validating the research hypothesis.

**Table 2:** Means, standard deviations, reliabilities, and correlations.

Variable	M	SD	1	2	3	4	5	6	7	8
(1) Gender	-	-	1							
(2) Age	1.62	0.49	0.05	1						
(3) Education	1.52	0.50	0.05	−0.70^**^	1					
(4) Challenge stressors	3.31	0.59	−0.07	−0.05	−0.04	1				
(5) Hindrance stressors	2.86	0.62	−0.08	−0.01	−0.09	0.33^**^	1			
(6) Job crafting	3.80	0.39	−0.17^**^	−0.12	0.01	0.25^**^	−0.14^**^	1		
(7) Leader-member exchange	3.68	0.52	−0.10	−0.15^*^	0.11	0.13^*^	−0.14^*^	0.45^**^	1	
(8) Organizational socialization	3.82	0.39	−0.15^*^	−0.04	−0.05	0.19^*^	−0.22^*^	0.54^*^	0.53^*^	1

M is the mean value, SD is the standard deviation. Age: born from 1990 to 1995 = 1, born after1995 = 2; Gender: male = 1, female = 2; Education level: bachelor’s degree or below = 1, master’s degree or above = 2.

* *p* < 0.05;

** *p* < 0.01.

### The mediating role of job crafting

This study used the PROCESS program’s hierarchical regression and bootstrap test to test the relationship between challenge stressors, hindrance stressors, and organizational socialization. After controlling for demographic variables, the regression model fitting results show that the regression models of challenge stressors (*R^2^* = 0.03, *F* = 8.74, *p* < 0.01) and hindrance stressors (*R^2^* = 0.05, *F* = 11.92, *p* < 0.01) passed the test. The regression coefficient of challenge stressors on organizational socialization was significant (*β* = 0.17, *p* < 0.01). At the same time, the regression coefficient of hindrance stressors on organizational socialization was also significant (*β* = −0.24, *p* < 0.001). The results showed that challenge stressors positively promoted the organizational socialization of newcomers, and hindrance stressors negatively promoted organizational socialization of newcomers, supporting the hypotheses H1a and H1b. After controlling for demographic variables, challenge stressors had a significant positive effect on job crafting (*β* = 0.23, *p* < 0.001), and hindrance stressors had a significant negative effect on job crafting (*β* = −0.17, *p* < 0.01). Therefore, the research hypotheses H2a and H2b were supported.

After that, challenge stressors and job crafting were added to the regression equation of organizational socialization, as shown in Table 3. The results showed that job crafting had a significant effect on organizational socialization (*β* = 0.51, *p* < 0.001), while challenge stressors had no significant effect on organizational socialization (*β* = 0.05, *p* > 0.05). This result suggests that job crafting mediates the effect of challenge stressors on organizational socialization. Thereby, hypothesis H2a was supported. The model 4 in PROCESS program was used to test the mediation effect of job crafting. The results of bootstrap method showed that the goodness of fit of the mediation effect was good (*R^2^* = 0.05, *F* = 3.45, *df_1_* = 4, *df_2_* = 242, *p* < 0.01). The total effect of challenge stressors on organizational socialization was significant (*β* = 0.11, *p* < 0.01, 95% *CI* [0.03, 0.19]), while the direct effect was not significant (*β* = 0.03, *p* > 0.05, 95% *CI* [−0.03, 0.10]). The indirect effect of job crafting between challenge stressors and organizational socialization was significant (*β* = 0.07, 95% *CI* [0.03, 0.13]). The results suggested that job crafting plays a complete mediating role between challenge stressors and organizational socialization, supporting hypothesis H3a.

**Table 3:** Results of multiple regression analysis.

Variable	Job crafting				Organizational socialization			
	Model 1	Model 2	Model 3	Model 4	Model 5	Model 6	Model 7	Model 8
Age	−0.16	−0.13	−0.09	−0.09	−0.07	0.01	−0.04	0.02
Gender	−0.15^*^	−0.14^*^	−0.10	−0.10	−0.14^*^	−0.05	−0.13^*^	−0.05
Education	−0.08	−0.04	−0.07	−0.06	−0.05	−0.01	−0.02	−0.01
Challenge stressors		0.23^***^	0.18^**^	0.18^**^			0.17^**^	0.05
Job crafting						0.53^***^		0.51^***^
Leader-member exchange			0.40^***^	0.41^***^				
Job crafting × leader-member exchange				0.12^*^				
*R^2^*	0.04	0.10	0.25	0.27	0.02	0.29	0.05	0.29
*ΔR^2^*	0.04^*^	0.10^***^	0.25^***^	0.27^***^	0.02	0.29^***^	0.05^**^	0.29^***^
*F*	3.71^*^	6.42^***^	16.36^***^	14.78^***^	2.05	25.20^***^	2.73^**^	20.33^***^

* *p* < 0.05;

** *p* < 0.01;

*** *p* < 0.001.

Subsequently, this study added hindrance stressors and job crafting to the regression equation of organizational socialization, as shown in Table 4. The results showed that job crafting had a significant effect on organizational socialization (*β* = 0.50, *p* < 0.001), and hindrance stressors also had a significant effect on organizational socialization (*β* = −0.15, *p* < 0.01). The results suggested that job crafting partially mediates the effect of hindrance stressors on organizational socialization, and hypothesis H2b was supported. The model 4 in PROCESS program was used to test the mediation effect of job crafting. The results of bootstrap method showed that the mediation effect degree of fitting is good (*R^2^* = 0.08, *F* = 5.37, *df1* = 4, *df_2_* = 242, *p* < 0.001). The total effect of hindrance stressors on organizational socialization was significant (*β* = −0.15, *p* < 0.001, 95% *CI* [−0.22, −0.07]), and the direct effect was significant (*β* = −0.09, *p* < 0.01, 95% *CI* [− 0.16, −0.02]). The indirect effect of job crafting between hindrance stressors and organizational socialization was significant (*β* = −0.05, 95% *CI* [−0.09, −0.01]). The results indicated that job crafting partially mediated hindrance stressors and organizational socialization, and hypothesis H3b was supported.

**Table 4:** Results of multiple regression analysis.

Variable	Job crafting				Organizational socialization			
	M1	M2	M3	M4	M5	M6	M7	M8
Age	−0.16	−0.19	−0.14	−0.14	−0.07	0.01	−0.11	−0.01
Gender	−0.15^*^	−0.16^*^	−0.12^*^	−0.12^*^	−0.14^*^	−0.05	−0.15^*^	−0.07
Education	−0.08	−0.12^*^	−0.11	−0.12	−0.05	−0.01	−0.11	−0.05
Hindrance stressors		−0.17^**^	0.11	−0.11			−0.24^***^	−0.15^**^
Job crafting						0.53^***^		0.50^***^
Leader-member exchange			0.41^***^	0.41^***^				
Job crafting × leader-member exchange				−0.03				
*R^2^*	0.04	0.07	0.23	0.23	0.02	0.29	0.08	0.31
*ΔR^2^*	0.04^*^	0.07^**^	0.23^***^	0.23^***^	0.02	0.29^***^	0.08^***^	0.31^***^
*F*	3.71^*^	4.76^**^	14.69^***^	14.78^***^	2.05	25.20^***^	5.37^***^	20.33^***^

* *p* < 0.05;

** *p* < 0.01;

*** *p* < 0.001.

### Validity of moderating and mediating effects

**Figure 2:**
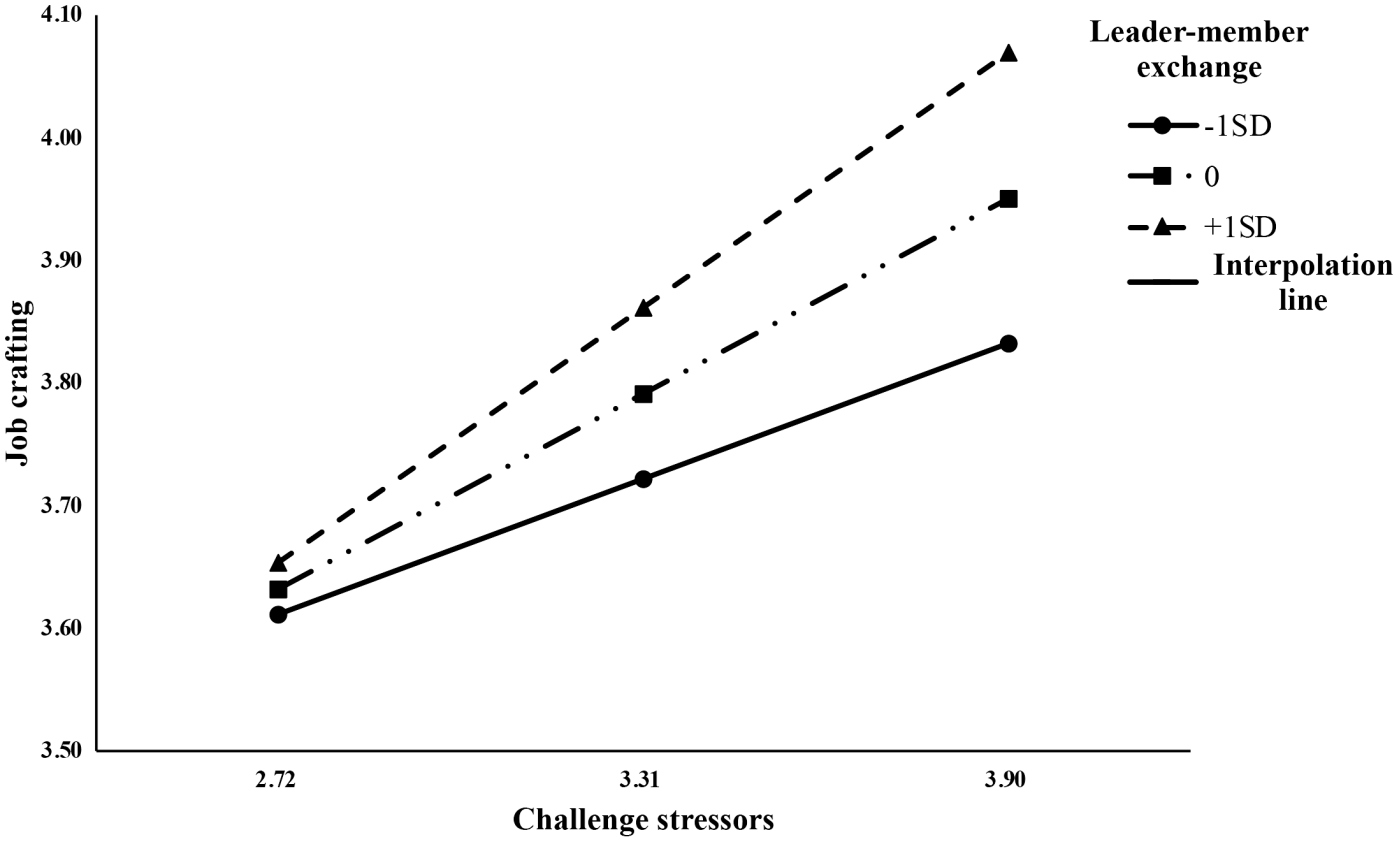
Interactive effect of challenge stressors and leader-member exchange on newcomers’ job crafting.

Hierarchical regression and the bootstrap method of the PROCESS procedure were used in this study to test moderated mediation models. The interaction term of challenge stressors and leader-member exchange has a significant positive predictive effect on job crafting (*β* = 0.12, *p* < 0.01; *ΔR^2^* = 0.27, *p* < 0.001; Table 3). This result indicates that the leader-member exchange has a moderating effect, which means that a higher leader-member exchange relationship under challenge stressors will show more job crafting. The model 7 in PROCESS program was used to test the moderating effect of leader-member exchange. Bootstrap results show that the mediating effect of job crafting between challenge stressors and organizational socialization varies with the level of leader-member exchange, and the moderated mediating effect is significant (*β* = 0.15, 95% *CI* [0.02, 0.28]). At the high level of leader-member exchange (one standard deviation above the mean), the mediating effect of job crafting between challenge stressors and organizational socialization (*β* = 0.10, 95% *CI* [0.04, 0.16]) was stronger than that at the low level of leader-member exchange (one standard deviation below the mean; *β* = 0.01, 95% *CI* [−0.03, 0.07]). The relationship between challenge stressors and job crafting made by taking high-level leader-member exchange and low-level leader-member exchange further verifies this relationship. Following Aiken and West (1991), we plotted the interactions at −1SD, 0, and + 1*SD* of leader-member exchange (Figure 2). In the case of high leader-member exchange, the slope of challenge stressors to job crafting is higher than that of low leader-member, indicating that the increase of leader-member exchange promotes the positive effect of challenge stressors on job crafting function. Thus, H4a was supported.

The interaction term of hindrance stressors and leader-member exchange had no significant effect on the prediction of job crafting (*β* = −0.03, *p* > 0.05; ΔR^2^ = 0.23, *p* < 0.001) (Table 4). This result indicated that the regulatory effect of leader-member exchange was not significant.

## Discussion

### Theoretical implications

Firstly, based on the theory of dual work stress, this study improved research on organizational socialization. As mentioned earlier, studies have focused more on the relationship between a particular type of stress or generalized work stress in a specific organizational behavior that is closely related to organizational socialization outcomes (e.g., Liu and Li, 2018; Zhou and Ning, 2020; Li et al., 2021), and less on how stress works from the perspective of categorical stress. This study divides stress into challenge stressors and hindrance stressors and explores their effects on the organizational socialization of newcomers. It is found that challenge stressors have a significant positive impact on organizational socialization of newcomers, while hindrance stressors have a significant negative effect on the organizational socialization of newcomers. Overall, the present study confirms the feasibility of the dichotomy classification of stressors faced by newcomers (O'Brien and Beehr, 2019) and provides theoretical support for future discussions of newcomers’ work psychology and behavior based on the challenge-hindrance stressor framework. In addition, empirical research on the impact of stress on newcomers’ organizational socialization has been advanced in this study. Previous studies on the impact of work stress on organizational socialization have mainly focused on theoretical discussions and rarely differentiated the impact mechanisms of different types of stress empirically. Based on the theory of Ellis et al. (2015), the stressor of entering a new environment is an important reason newcomers fail to develop a positive attitude toward their new organization, but relevant empirical evidence is lacking. This study found differences in the impact of different types of stress on organizational socialization. It was found that not all job stressors lead to negative organizational socialization outcomes for newcomers and that challenge stressors positively affect newcomers’ organizational socialization. The results advanced the assumption of Ellis et al.

Second, this study illustrates the mechanisms that transform newcomers from “outside members” to “inside members” in different types of stressful situations. Based on job-crafting theory, this study explores the mechanisms of organizational socialization through autonomously changing and shaping work tasks, relationships, and cognitions after newcomers enter a new work environment. The findings support the notion that employees engage in job crafting early in their careers (Fried et al., 2007) and find that job crafting has different mediation effects in different stressful situations. Specifically, job crafting plays a fully mediating role under challenge stressors, while in hindrance stressors, job crafting plays a partially mediating role. We believe that the source of challenge stressors is the pressure that can help newcomers to improve their workability and gain future benefits, which will directly activate their motivation to overcome the pressure by various means, i.e., “pressure is motivation, “as the saying goes. At the same time, in the existing job-crafting framework, increasing challenging job demands is an important part of employee job crafting (Tims et al., 2012). In other words, newcomers can use job crafting to completely overcome the pressure and facilitate their adaptation to the new job when they facing challenge stressors. Namely, job crafting fully mediates the relationship between challenge stressors and organizational socialization. Whereas hindrance stressors can directly hinder newcomers’ work, the negative effects of such stressors can only be mitigated to some extent by job crafting. Therefore, job crafting partially mediates the relationship between hindrance stressors and organizational socialization.

Third, this study demonstrated the JD-R theory for newcomers’ organizational socialization. According to the JD-R model, high job demands and low job resources trigger job burnout or turnover, but increasing challenging job demands and resources leads to better job outcomes (Bakker and Demerouti, 2007). High-quality LMX facilitates newcomers’ access to more job resources (Graen and Cashman, 1975; Graen and Uhl-Bien, 1995). The results supported that LMX is an important moderator of newcomers’ socialization (Yao and Yue, 2011). It showed that challenge stressors positively relate to newcomers’ organizational socialization *via* job crafting under high LMX. However, LMX did not moderate the relationship between hindrance stressors, job crafting, and organizational socialization. The results of the present study are not consistent with the findings of Spurk et al. (2021). Their study found that LMX was able to moderate the process of CPC as a challenge versus a hindrance to job requirements for subjective and objective career success. In the present study, we suggest that the result difference may be the LMX as a job resource for newcomers. There are differences in the mechanisms by which it interacts with different stressors/demands. Compared to low LMX conditions, high LMX conditions allow newcomers to have sufficient or even redundant work resources to face the high sources/demands of challenge stressors they encounter, increasing their space for self-expression and autonomous action, which, in turn, leads to better organizational socialization results. However, in high LMX conditions, although leaders give newcomers more resources, these resources do not necessarily directly affect overcoming hindrance stressors/demands. For example, the organizational policies as hindrance stressors may have the same impact on the leader and the newcomer. Even if the newcomer uses the resources obtained from high LMX for job crafting to increase their adaptation to the new job, they may not necessarily receive the desired results. This can lead to newcomers not using the job resources to enhance their job crafting even if they have sufficient job resources from leaders. This confirms the JD-R theory that the interaction between specific job demands and specific job resources affects employees’ work behavior (Bakker and Demerouti, 2007). Another possibility is that the stressors are categorized in different ways. Our study’s challenge and hindrance stressors are completely different categories of stressors (O'Brien and Beehr, 2019). However, in their study, although CPC was defined as challenge and hindrance demands by different appraisal path, it was essentially the same stressor. They argue that examining stressors using a categorical approach (this study’s approach) versus an assessment approach (their study) has much to discuss. However, since this is not a topic for an in-depth discussion in this study, it could be a direction of interest for future research.

### Practical implications

The findings of this paper have some practical implications for newcomers to adapt to the new environment. First of all, managers should classify and manage the work requirements of newcomers according to the different types of stressors. According to the findings, stressors would produce different organizational socialization outcomes. Therefore, on the one hand, managers have to increase the demands of work characterized by the challenge stressors. When newcomers face the requirements of the challenge work stressors, rewarding work experience will make individuals feel that they are trusted and are competent for the job. This results in positive organizational behavior (Chen et al., 2021), such as building better interpersonal relationships, enhancing job competency, and better integrating into the new organization. On the other hand, managers should reduce the work demands characterized by hindrance stressors to mitigate their negative impact on the socialization of newcomers in organizations.

Secondly, in the process of organizational socialization, managers should pay attention to the initial behavior of newcomers to adjust the work content. Research showed that the new generation of employees are more self-oriented and pursue autonomy (Li and Hou, 2012). This study also found that different stressors can improve the job crafting of newcomers, which help strengthen their organizational socialization and promote their efficient integration into the organization. Therefore, in the process of newcomers adapting to the organization, managers can provide a good organizational support environment to encourage newcomers to do job crafting, for example, improving the degree of freedom and work independence (Tims and Bakker, 2010), developing a job reshaping instruction manual, organizing systematic job reshaping training, etc. Wei et al. (2018) believed that job crafting might be an important key to unlocking the management problems of the new generation of employees.

Finally, organizational socialization strategies should be adapted to the characteristics of newcomers. Kehrli and Sopp (2006) argued that the emphasis on rational organizational rules is no longer sufficient to meet the psychological needs of newcomers, who place more emphasis on a human-centered atmosphere. Therefore, (1) organizations should encourage newcomers to participate in the development of work programs and fully respect their opinions to take advantage of their professional autonomy and maximize the effectiveness of both newcomers and the organization; (2) foster a supportive leadership style, help employees to self-direct and manage, and present a clear vision of development to stimulate more proactive behaviors. Leaders can facilitate newcomers’ job crafting by building and maintaining a high LMX, encouraging newcomers’ organizational socialization; and (3) consider the differences in newcomers’ proactive behaviors and adopt a differentiated organizational socialization strategy.

## Limitations and future research

Although the results of this study have significant academic potential and policy implications, there are still some areas to be improved. Firstly, the adaptation of newcomers to the new work environment is a dynamic and gradual process. Some bias may exist as the study uses cross-sectional data to infer the impact of challenge-hindrance stressors on organizational socialization. Future research can adopt a longitudinal design to collect employee behavior data at different time points to explore the causal relationship between variables more accurately. Secondly, this paper took the proximal outcome of organizational socialization as the research variable. In the future, we can also study distant outcome indicators such as job satisfaction, organizational commitment, and turnover intention and further explore the impact of stress factors in work scenarios on organizational socialization.

## Data availability statement

The raw data supporting the conclusions of this article will be made available by the authors, without undue reservation.

## Ethics statement

Ethical review and approval was not required for the study on human participants in accordance with the local legislation and institutional requirements. The patients/participants provided their written informed consent to participate in this study.

## Author contributions

YT and ZZ conceived the project, designed the study, and oversaw the whole research process. JZ was responsible for data collection and data analysis. SW was responsible for drafting the manuscript and final approval of the manuscript. All authors contributed to the article and approved the submitted version.

## Funding

This study was supported by the National Social Science Foundation of China (grant no. 21XDJ002).

## Publisher’s note

All claims expressed in this article are solely those of the authors and do not necessarily represent those of their affiliated organizations, or those of the publisher, the editors and the reviewers. Any product that may be evaluated in this article, or claim that may be made by its manufacturer, is not guaranteed or endorsed by the publisher.

## Conflict of interest

The authors declare that the research was conducted in the absence of any commercial or financial relationships that could be construed as a potential conflict of interest.
